# The significance of isolated de novo red patches in the bladder in patients referred with suspected urinary tract cancer: Results from the IDENTIFY study

**DOI:** 10.1002/bco2.475

**Published:** 2025-01-06

**Authors:** Sinan Khadhouri, Kevin Gallagher, Kenneth R. MacKenzie, Taimur T. Shah, Chuanyu Gao, Eleanor Zimmermann, Miles Mannas, Taeweon Lee, Giancarlo Marra, Juan Gomez Rivas, Gautier Marcq, Mark A. Assmus, Taha Ucar, Francesco Claps, Matteo Boltri, Giuseppe Pizzuto, Tara Burnhope, Nkwam Nkwam, George Tanasescu, Nicholas E. Boxall, Alison P. Downey, Troy A. Sukhu, Marta Antón‐Juanilla, Sonpreet Rai, Madeline Moore, Kathryn Bandeira de Mello, Sian Parsons, John S. McGrath, Veeru Kasivisvanathan, IDENTIFY Study Group: Pubmed indexed collaborator authors, Aasem Chaudry, Abhishek Sharma, Adam Bennett, Adnan Ahmad, Ahmed Abroaf, Ahmed Musa Suliman, Aimee Lloyd, Alastair McKay, Albert Wong, Alberto Silva, Alexandre Schneider, Alison MacKay, Allen Knight, Alkiviadis Grigorakis, Amar Bdesha, Amy Nagle, Ana Cebola, Ananda Kumar Dhanasekaran, Andraž Kondža, André Barcelos, Andrea Benedetto Galosi, Andrea Ebur, Andrea Minervini, Andrew Russell, Andrew Webb, Ángel García de Jalón, Ankit Desai, Anna Katarzyna Czech, Anna Mainwaring, Anthony Adimonye, Arighno Das, Arjun Nambiar, Arnaldo Figueiredo, Arnauld Villers, Artur Leminski, Arvinda Chippagiri, Asim Ahmed Lal, Asıf Yıldırım, Athanasios Marios Voulgaris, Audrey Uzan, Aye Moh Moh Oo, Ayman Younis, Bachar Zelhof, Bashir Mukhtar, Ben Ayres, Ben Challacombe, Benedict Sherwood, Benjamin Ristau, Billy Lai, Brechtje Nellensteijn, Brielle Schreiter, Carlo Trombetta, Catherine Dowling, Catherine Hobbs, Cayo Augusto Estigarribia Benitez, Cédric Lebacle, Cherrie Wing Yin Ho, Chi‐Fai Ng, Chloe Mount, Chon Meng Lam, Chris Blick, Christian Brown, Christopher Gallegos, Claire Higgs, Clíodhna Browne, Conor McCann, Cristina Plaza Alonso, Daniel Beder, Daniel Cohen, Daniel Gordon, Daniel Wilby, Danny Gordon, David Hrouda, David Hua Wu Lau, Dávid Karsza, David Mak, David Martin‐Way, Denula Suthaharan, Dhruv Patel, Diego M. Carrion, Donald Nyanhongo, Edward Bass, Edward Mains, Edwin Chau, Elba Canelon Castillo, Elizabeth Day, Elsayed Desouky, Emily Gaines, Emma Papworth, Emrah Yuruk, Enes Kilic, Eoin Dinneen, Eric Edison, Erika Palagonia, Evanguelos Xylinas, Faizan Khawaja, Fernando Cimarra, Florian Bardet, Francesca Kum, Francesca Peters, Gábor Kovács, Geroge Tanasescu, Giles Hellawell, Giovanni Tasso, Gitte Lam, Giuseppe La Montagna, Giuseppe Pizzuto, Gordan Lenart, Graeme MacLennan, Günal Özgür, Hai Bi, Hannah Lyons, Hannah Warren, Hashim Ahmed, Helen Simpson, Helena Burden, Helena Gresty, Hernado Rios Pita, Holly Clarke, Hosam Serag, Howard Kynaston, Hugh Crawford‐Smith, Hugh Mostafid, Hugo Otaola‐Arca, Hui Fen Koo, Ibrahim Ibrahim, Idir Ouzaid, Ignacio Puche‐Sanz, Igor Tomašković, Ilker Tinay, Iqbal Sahibzada, Isaac Thangasamy, Iván Revelo Cadena, Jacques Irani, Jakub Udzik, James Brittain, James Catto, James Green, James Tweedle, Jamie Borrego Hernando, Jamie Leask, Jas Kalsi, Jason Frankel, Jason Toniolo, Jay D. Raman, Jean Courcier, Jeevan Kumaradeevan, Jennifer Clark, Jennifer Jones, Jeremy Yuen‐Chun Teoh, John Iacovou, John Kelly, John P. Selph, Jonathan Aning, Jon Deeks, Jonathan Cobley, Jonathan Olivier, Jonny Maw, José Antonio Herranz‐Yagüe, Jose Ignacio Nolazco, Jose Manuel Cózar‐Olmo, Joseph Bagley, Joseph Jelski, Joseph Norris, Joseph Testa, Joshua Meeks, Juan Hernandez, Juan Luis Vásquez, Karen Randhawa, Karishma Dhera, Katarzyna Gronostaj, Kathleen Houlton, Kathleen Lehman, Kathryn Bandeira de Mello, Kelvin Adasonla, Kevin Brown, Kevin Murtagh, Kiki Mistry, Kim Davenport, Kosuke Kitamura, Laura Derbyshire, Laurence Clarke, Lawrie Morton, Levin Martinez, Louise Goldsmith, Louise Paramore, Luc Cormier, Lucio Dell'Atti, Lucy Simmons, Luis Martinez‐Piñeiro, Luis Rico, Luke Chan, Luke Forster, Lulin Ma, Madeline Moore, Maria Camacho Gallego, Maria José Freire, Mark Emberton, Mark Feneley, Marta Antón‐Juanilla, Marta Viridiana Muñoz Rivero, Matea Pirša, Matteo Tallè, Matthew Crockett, Matthew Jefferies, Matthew Liew, Matthew Nielsen, Matthew Trail, Meghan Cooper, Meghana Kulkarni, Michael Ager, Ming He, Mo Li, Mohamed Omran Breish, Mohamed Tarin, Mohammed Aldiwani, Mudit Matanhelia, Muhammad Pasha, Mustafa Kaan Akalın, Nasreen Abdullah, Nathan Hale, Neha Gadiyar, Neil Kocher, Nicholas Bullock, Nicholas Campain, Nicola Pavan, Nihad Al‐Ibraheem, Nikita Bhatt, Nishant Bedi, Nitin Shrotri, Niyati Lobo, Olga Balderas, Omar Kouli, Otakar Capoun, Pablo Oteo Manjavacas, Paolo Gontero, Paramananthan Mariappan, Patricio Garcia Marchiñena, Paul Erotocritou, Paul Sweeney, Paula Planelles, Peter Acher, Peter C. Black, Peter K. Osei‐Bonsu, Peter Østergren, Peter Smith, Peter‐Paul Michiel Willemse, Piotr L. Chlosta, Qurrat Ul Ain, Rachel Barratt, Rachel Esler, Raihan Khalid, Ray Hsu, Remigiusz Stamirowski, Reshma Mangat, Ricardo Cruz, Ricky Ellis, Robert Adams, Robert Hessell, Robert J. A. Oomen, Robert McConkey, Robert Ritchie, Roberto Jarimba, Rohit Chahal, Rosado Mario Andres, Rosalyn Hawkins, Rotimi David, Rustom P. Manecksha, Sacha Moore, Sachin Agrawal, Syed Sami Hamid, Samuel Deem, Sanchia Goonewardene, Satchi Kuchibhotla Swami, Satoshi Hori, Shahid Khan, Shakeel Mohammud Inder, Shanthi Sangaralingam, Shekhar Marathe, Sheliyan Raveenthiran, Shigeo Horie, Shomik Sengupta, Sian Parson, Sidney Parker, Simon Hawlina, Simon Williams, Simone Mazzoli, Slawomir Grzegorz Kata, Sofia Pinheiro Lopes, Sónia Ramos, Sonpreet Rai, Sophie Rintoul‐Hoad, Sorcha O'Meara, Steve Morris, Stacey Turner, Stefano Venturini, Stephanos Almpanis, Steven Joniau, Sunjay Jain, Susan Mallett, Sven Nikles, Syed Shahzad, Sylvia Yan, Taeweon Lee, Taha Uçar, Tamsin Drake, Tarq Toma, Teresa Cabañuz Plo, Thierry Bonnin, Tim Muilwijk, Tim Wollin, Timothy Shun Man Chu, Timson Appanna, Tom Brophy, Tom Ellul, Tomas Austin, Tomaž Smrkolj, Tracey Rowe, Troy Sukhu, Trushar Patel, Tullika Garg, Turhan Çaşkurlu, Uros Bele, Usman Haroon, Víctor Crespo‐Atín, Victor Parejo Cortes, Victoria Capapé Poves, Vincent Gnanapragasam, Vineet Gauhar, Vinnie During, Vivek Kumar, Vojtech Fiala, Wasim Mahmalji, Wayne Lam, Yew Fung Chin, Yigit Filtekin, Yih Chyn Phan, Youssed Ibrahim, Zachary A. Glaser, Zainal Adwin Abiddin, Zijian Qin, Zsuzsanna Zotter, Zulkifli Zainuddin

**Affiliations:** ^1^ University of St Andrews St Andrews United Kingdom; ^2^ Western General Hospital Edinburgh United Kingdom; ^3^ South Tyneside and Sunderland NHS Foundation Trust Sunderland United Kingdom; ^4^ Imperial College London London United Kingdom; ^5^ Addenbrookes Hospital Cambridge United Kingdom; ^6^ University Hospitals Plymouth Plymouth United Kingdom; ^7^ Department of Urologic Sciences University of British Columbia Vancouver Canada; ^8^ Department of Surgical Sciences, Division of Urology, Città della Salute e della Scienza University of Turin Turin Italy; ^9^ Department of Urology La Paz University Hospital Madrid Spain; ^10^ Urology Department Claude Huriez Hospital, CHU Lille Lille France; ^11^ CNRS, Inserm, CHU Lille, Institut Pasteur de Lille, UMR9020‐U1277 – CANTHER – Cancer Heterogeneity Plasticity and Resistance to Therapies University Lille Lille France; ^12^ Section of Urology, Department of Surgery University of Calgary Calgary Alberta Canada; ^13^ Private Medicabil Hospital Department of Urology Bursa Turkey; ^14^ Urological Clinic, Department of Medicine, Surgery and Health Sciences University of Trieste Trieste Italy; ^15^ Department of Surgery, Division of Urology San Giovanni di Dio Hospital Gorizia Italy; ^16^ University Hospitals Birmingham NHS Foundation Trust Birmingham United Kingdom; ^17^ University Hospitals of Derby and Burton NHS Foundation Trust Derby United Kingdom; ^18^ Department of Urology Queen Alexandra Hospital Portsmouth United Kingdom; ^19^ Salford Royal NHS Foundation Trust Salford United Kingdom; ^20^ Doncaster Royal Infirmary Doncaster United Kingdom; ^21^ University of North Carolina Hospitals Chapel Hill NC USA; ^22^ Department of Urology Hospital Universitario Cruces Barakaldo Spain; ^23^ Milton Keynes University Hospital NHS Foundation Trust Milton Keynes United Kingdom; ^24^ Royal Devon and Exeter NHS Foundation Trust Exeter United Kingdom; ^25^ Southmead Hospital Bristol United Kingdom; ^26^ University of Exeter Medical School Exeter United Kingdom; ^27^ University College London London United Kingdom

**Keywords:** biopsy, bladder cancer, cystoscopy, haematuria, red patch, risk factors

## Abstract

**Objectives:**

To assess the contemporary malignancy rate in isolated de novo red patches in the bladder and associated risk factors for better selection of red patch biopsy.

**Patients:**

Patients from the IDENTIFY dataset; Patients referred to secondary care with suspected urinary tract cancer and found to have isolated de novo red patches on cystoscopy.

**Methods:**

We reported the unadjusted cancer prevalence in isolated de novo red patches that were biopsied; multivariable logistic regression was used to explore cancer‐associated risk factors including age, sex, smoking, type of haematuria, LUTS, UTIs and a suspicious‐looking red patch (as reported by the cystoscopist). Sub‐analysis of these by clinical role and experience was performed.

**Results:**

A total of 1110 patients with isolated de novo red patches were included. 41.5% (n = 461) were biopsied, with a malignancy rate of 12.8% (59/461), which was significantly higher in suspicious versus non‐suspicious red patches (19.1% vs. 2.81%, p < 0.01). There was a significant association between bladder cancer and age (OR 1.04, 95% CI 1.01–1.07, p = 0.01), smoking history (OR 2.62, 95% CI 1.09–6.27, p = 0.03) and suspicious‐looking patch (OR 6.50, 95% CI 2.47–17.1, p < 0.01). The majority of malignancies were in over 60‐year‐olds. Malignancy rates in suspicious versus non‐suspicious red patches did not differ significantly between clinical roles or experiences.

Limitations included subjectivity in classifying a suspicious patch and selection bias as not all patches were biopsied.

**Conclusions:**

Many patients still undergo unnecessary biopsies under general anaesthetic for isolated de novo red patches. Clinicians should consider the patient's age, smoking status and how suspicious‐looking the patch is, before deciding on surveillance versus biopsy to improve cancer diagnostic yield.

## 1 INTRODUCTION

Isolated de novo red patches in the bladder (defined as new red patches without a prior history of urothelial cancer) are often biopsied to determine if they are benign or malignant. These are usually found when patients undergo a cystoscopy for suspected urinary tract cancer. Most evidence regarding the outcome of red patch biopsies relates to patients with a known history of bladder cancer undergoing surveillance.^1^ The limited data on de novo red patches in patients with haematuria comes from small single‐centre studies that report malignancy in 10–18%.^2^, ^3^ This implies that over 80% of patients have an unnecessary invasive procedure, posing a large burden on health resources and exposing patients to unnecessary associated risk including infection. Risk factors associated with a malignant red patch are considered in these studies to better select patients, such as urinary tract infections (UTIs) and lower urinary tract symptoms (LUTS). However, they fail to include well‐known risk factors for bladder cancer such as type of haematuria, smoking, age and sex.^4^ Furthermore, there is no consideration of how suspicious‐looking a red patch is at cystoscopy, nor the expertise of the clinician performing the cystoscopy.

Concomitant red patches with urothelial carcinoma in the bladder are often used to predict the recurrence and progression of the disease and are treated separately to patients with isolated de novo red patches.^5^, ^6^

The IDENTIFY study is the largest multinational prospective study on patients referred to secondary care with suspected urinary tract cancer.^7^

In this analysis of the IDENTIFY dataset, we aim to determine outcomes of isolated de novo red patch biopsy and risk factors associated with malignancy in these.

## 2 PATIENTS AND METHODS

### 2.1 Study design and setting

This study is a sub‐analysis of the main IDENTIFY study, which was a prospective international multicentre study and included 26 countries, 110 centres.^8^ Data was collected prospectively on patients referred to secondary care for a diagnostic cystoscopy because of suspected urinary tract cancer between December 2017 and December 2018. Patients were followed up until their investigations were concluded and a diagnosis confirmed or ruled out, as per the judgement of the clinical care team. The study was closed in February 2019.

### 2.2 Participants

The inclusion criteria for the IDENTIFY study were patients aged 16 years or over, with or without haematuria, referred to a urologist for the investigation of suspected urinary tract cancer, and without a previous or known diagnosis of primary urological cancer. For this analysis, patients were included if they had an isolated *de‐novo* red patch and were excluded if they had a concomitant suspected bladder tumour.

### 2.3 Data collection

Data collected included baseline demographics, investigation findings and histopathology from biopsies.^8^ Red patches were defined as an abnormal discoloured area in the bladder and were categorised into suspicious or non‐suspicious (for malignancy), as reported by the cystoscopist. Suspicious red patches had appearances more typical of carcinoma‐in‐situ (CIS) such as a velvety patch of erythematous mucosa. Non‐suspicious red patches had appearances more typical of inflammation such as erythematous bullous mucosa or petechial patches. The type of haematuria was determined by the primary care referral letter and/or the history of the patients at the time of assessment in secondary care. Non‐visible haematuria was defined by a trace or more on a urine dipstick, or over three red blood cells per high‐power field. Smoking status was categorised into current smoker, ex‐smoker and never smoked. UTIs were categorised as single or recurrent (two or more infections in 6 months or 3 or more infections in 1 year) according to patient history and culture results. The role and experience (number of cystoscopies performed) of the clinician doing the cystoscopy were collected for each patient case.

### 2.4 Outcomes

The primary outcome was the proportion of biopsied isolated de novo red patches that were confirmed to be malignant on pathological analysis.

### 2.5 Risk factors/predictors

Factors assessed for association with malignancy in biopsied red patches were: suspicious reported red patch, age, sex, smoking, type of haematuria, LUTS and UTIs. Separately, the association of cystoscopic experience and clinical role with malignancy in suspicious reported red patches and non‐malignancy in non‐suspicious reported red patches were assessed.

### 2.6 Diagnostic accuracy of cytology

The diagnostic accuracy of cytology in red patches for malignancy found at biopsy, stratified by suspicious and non‐suspicious reported red patches will be reported.

### 2.7 Statistical analysis

Proportions were calculated as the target outcome divided by the target population. The analysis of red patches was at the patient level, not the lesion level. The association of patient risk factors with malignant red patches were analysed using a multivariable logistic regression model. The variables were chosen based on previous evidence, clinical judgement and those with a biological plausibility for having an association with bladder cancer.^7^ Diagnostic accuracy of cytology was calculated as sensitivity, specificity and negative and positive predictive values. Two‐sample test of proportions was used to compare the different diagnostic accuracies between suspicious and non‐suspicious reported red patches. All analyses were performed using Stata version 16.1 (StataCorp, College Station, Texas, United States). A p‐value of less than 0.05 was deemed statistically significant.

## 3 RESULTS

**FIGURE 1:**
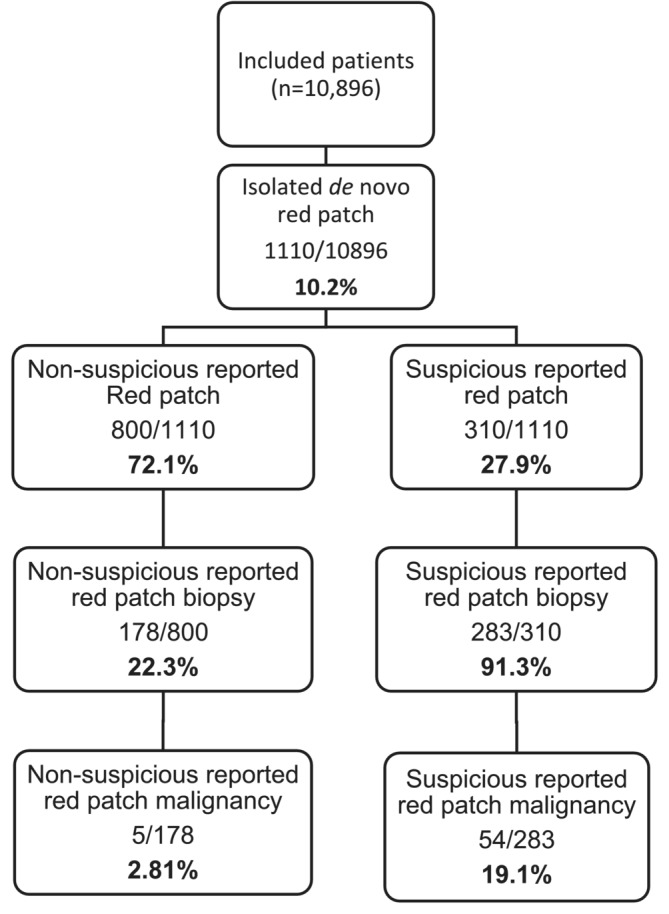
Cohort flow diagram of patients found to have an isolated de novo red patch.

There were a total of 1110 (10.2%) patients with isolated de novo red patches found on flexible cystoscopy in a cohort of 10 896 (Figure 1). The majority of these (72.1%, n = 800/1110) were reported to be non‐suspicious by the cystoscopist. A total of 461/1110 (41.5%) red patches were biopsied. There was a greater proportion of biopsies taken from patients with suspicious red patches compared to non‐suspicious red patches (91.3% vs. 22.3%). Overall, the malignancy rate from all biopsied red patches was 12.8% (59/461). The malignancy rate in suspicious red patches was higher than in non‐suspicious red patches (19.1% vs. 2.81%). Out of the 59 malignancies found, 50 (84.7%) were urothelial cancer, seven (11.9%) were adenocarcinoma and two (3.39%) were squamous cell carcinoma. Isolated CIS accounted for over half (55.1%) of the urothelial cancers; the rest were high‐grade diseases (24.5%) and low‐grade diseases (20.4%).

Table 1 shows the patient characteristics and multivariable analysis of biopsied isolated de novo red patches. Significant cancer‐associated risk factors were: a suspicious reported red patch (OR 6.50, 95% CI 2.47–17.1, p < 0.001), age (OR 1.04, 95% CI 1.01–1.07, p = 0.01), ex‐smoker (OR 2.39, 95% CI 1.14–5.00, p = 0.02) and current smoker (OR 2.62, 95% CI 1.09–6.27, p = 0.03). The majority of cancers were in patients over the age of 60 years. Whilst there seemed to be a lower proportion of malignancy in those with a UTI, this did not meet the level of statistical significance on multivariable analysis.

**TABLE 1:** Patient characteristics of biopsied de novo red patches in malignant and non‐malignant red patches, with multivariable logistic regression analysis of patient risk factors.

	All biopsied de novo red patches (n = 461)		Multivariable analysis	
	Malignant (n = 59)	Non‐malignant (n = 402)	Odds ratio (95% CI)	p value
**Suspicious reported red patch, n (%)**				
No	5 (2.81)	173 (97.2)	1.00	
Yes	54 (19.1)	229 (80.9)	6.50 (2.47–17.1)	**<0.001**
**Age, n (%)**				
Mean (SD)	72.2 (9.84)	66.7 (13.5)	1.04 (1.01–1.07)	**0.01**
*≤30 years*	0 (0)	3 (100)		
*31–40 years*	0 (0)	16 (100)		
*41–50 years*	1 (3.2)	30 (96.8)		
*51–60 years*	6 (7.7)	72 (92.3)		
*61–70 years*	18 (13.5)	115 (86.5)		
*71–80 years*	22 (18.3)	98 (81.7)		
*≥80 years*	12 (15.0)	68 (85.0)		
**Type of haematuria, n (%)**				
No haematuria	4 (11.4)	31 (88.6)	1.00	
Non‐visible haematuria	12 (12.5)	84 (87.5)	1.50 (0.40–5.62)	0.55
Visible haematuria	4 (13.0)	287 (87.0)	1.16 (0.36–3.72)	0.81
**Sex, n (%)**				
Female	14 (7.41)	175 (92.6)	1.00	
Male	45 (16.6)	226 (83.4)	1.33 (0.63–2.82)	0.45
**Smoking, n (%)**				
Never smoked	18 (8.22)	201 (91.8)	1.00	
Ex‐smoker	23 (17.6)	108 (82.4)	2.39 (1.14–5.00)	**0.02**
Current smoker	14 (20.9)	53 (79.1)	2.62 (1.09–6.27)	**0.03**
**LUTS, n (%)**				
None	20 (9.39)	193 (90.6)	1.00	
Any LUTS	38 (15.6)	206 (84.4)	1.19 (0.62–2.28)	0.61
*Voiding LUTS*	*14 (20.3)*	*55 (79.7)*		
*Storage LUTS*	*14 (12.3)*	*100 (87.7)*		
*Mixed LUTS*	*10 (16.4)*	*51 (83.6)*		
**UTI History, n (%)**				
None	50 (13.4)	323 (86.6)	1.00	
Single	7 (8.54)	75 (91.5)	0.43 (0.15–1.19)	0.10
Recurrent	5 (7.46)	62 (92.5)	0.48 (0.15–1.55)	0.22

Percentages are row percentages; LUTS = Lower urinary tract symptoms; UTI = Urinary tract infection; CI = Confidence interval. Number of observations in multivariable logistic regression model = 452.

A total of 449/1110 (40.5%) patients who had a red patch had urine cytology tested. Of patients who had a biopsy, 39.9% (184/461) had urine cytology. Table 2a shows the diagnostic accuracy of cytology in patients who had a biopsy for a red patch, and Table 2b stratifies this by suspicious and non‐suspicious reported red patch. There was no significant difference for urine cytology in specificity, sensitivity and negative predictive value when comparing non‐suspicious versus suspicious reported red patches. However, the positive predictive value was significantly higher in suspicious compared to non‐suspicious reported red patches (53.8% vs. 8.3%, p < 0.01).

Cystoscopists with greater experience and more senior clinical roles seemed to have a higher prevalence of malignancy in their suspicious reported red patches, but this was not statistically significant (Table 3). There was also no significant difference in the prevalence of malignancy in non‐suspicious reported red patches between the two groups.

**TABLE 3:** Outcome of suspicious and non‐suspicious reported de novo red patch biopsy stratified by clinical role and experience in cystoscopy.

Experience (number of cystoscopies performed) n, (%)	Suspicious reported			Non‐suspicious reported		
	Malignant	Non‐malignant	Odds ratio of malignancy [95% CI]	Malignant	Non‐malignant	Odds ratio of malignancy [95% CI]
1 to 199	9 (15.0)	51 (85.0)	1.00	2 (6.25)	30 (93.8)	1.00
200 or more	44 (19.8)	178 (80.2)	1.40 [0.64–3.06] p = 0.40	3 (2.05)	143 (97.7)	0.31 [0.05–1.97] p = 0.22
**Clinical role, n (%)**						
Non‐Consultant/Attending	30 (16.8)	149 (83.2)	1.00	4 (3.74)	103 (96.3)	1.00
Consultant/Attending	24 (23.1)	80 (76.9)	1.49 [0.82–2.72] p = 0.19	1 (1.41)	70 (98.6)	0.37 [0.04–3.36] p = 0.38

Percentages are row percentages. For clinical roles, non‐consultant/attending constitutes trainee doctors, registrars/residents, other clinicians and urology nurses.

**TABLE 2a:** Diagnostic accuracy of cytology in patients found to have isolated de novo red patches.

		Bladder cancer		
		Negative	Positive	Total
Urine cytology	Negative	127	7	134
	Positive	28	22	50
	Total	155	29	184
Sensitivity % (95% CI)		75.9 (56.5–89.7)		
Specificity % (95% CI)		81.9 (75.0–87.6)		
Positive predictive value % (95% CI)		44.0 (30.0–58.7)		
Negative predictive value % (95% CI)		94.8 (89.5–97.9)		

A total of 184/461 (39.9%) patients who had a biopsied red patch had urine cytology tested. Positive urine cytology was defined as malignant or atypical cells/equivocal findings.

**TABLE 2b:** Diagnostic accuracy of cytology in patients found to have isolated de novo red patches stratified by suspicious reported and non‐suspicious reported red patches.

		Suspicious reported red patches			Non‐suspicious reported red patches			
		Bladder cancer			Bladder cancer			
		Negative	Positive	Total	Negative	Positive	Total	
Urine cytology	Negative	64	6	70	62	1	63	
	Positive	18	21	39	11	1	12	
	Total	82	27	109	73	2	75	
Sensitivity % (95% CI)		77.8 (57.7–91.4)			50.0 (1.3–98.7)			p = 0.38
Specificity % (95% CI)		78.0 (67.5–86.4)			84.9 (74.6–92.2)			p = 0.26
Positive predictive value % (95% CI)		53.8 (37.2–69.9)			8.3 (0.2–38.5)			p < 0.01
Negative predictive value % (95% CI)		91.4 (82.3–96.8)			98.4 (91.5–100)			p = 0.07

Positive urine cytology was defined as malignant or atypical cells/equivocal findings. Comparison of diagnostic accuracies was analysed using two‐sample test of proportions.

## 4 DISCUSSIONS

We present this secondary analysis from the IDENTIFY study, the largest international prospective observational study on the investigation of suspected urinary tract cancer in secondary care. This analysis characterises the significance of de novo red patches together with the risk factors associated with urological malignancy.

We report the proportion of malignancy in biopsied red patches to be 12.8%. This was higher in suspicious reported red patches (19.1%) than non‐suspicious reported red patches (2.81%). Although suspicion of a red patch is subjective, there is clearly value in a clinician making this differentiation. Despite this, however, the majority of patients do not have cancer and thus there is room for improvement in the selection of patients for biopsy of red patches. Bladder cancer was significantly associated with a suspicious reported red patch, age and smoking. Regarding cytology, specificity, sensitivity and negative predictive value were not significantly different in a suspicious reported red patch compared to a non‐suspicious red patch. Conversely, the positive predictive value was significantly higher in suspicious reported red patch compared to non‐suspicious, meaning a positive cytology confirms the need for urgent biopsy to assess for bladder cancer. There was no strong evidence to suggest more senior clinicians and those with more cystoscopic experience were better at reporting malignant red patches as suspicious, and benign red patches as non‐suspicious.

Our reported rate of malignancy in isolated de novo red patches (12.8%) is consistent with previous studies (10–18%).^2^, ^3^ Our study further stratifies this by suspicion of malignancy at cystoscopy, showing a significantly higher rate of malignancy in suspicious reported red patches (19.1%) versus non‐suspicious red patches (2.81%). Fernando et al. looked at the association of UTIs and LUTs with malignancy but did not find any CIS from red patch biopsies in patients with recurrent UTIs, however, 16% of their patients with LUTS had a malignant red patch.^2^ Our findings support this, with a lower proportion of malignancy in patients with UTI compared to those without (8.5% vs. 13.4%), and a higher proportion of malignancy in patients with LUTS (15.6% vs 9.4%), although these were not significant on multivariable analysis. Regarding age as a risk factor, one study of 193 red patch biopsies did not show any malignancy in patients under the age of 60 years.^3^ However, they had a heterogenous population sample containing patients with a previous diagnosis of urothelial cancer undergoing surveillance, and patients newly investigated for haematuria. Our results showed the youngest patient with a malignant isolated de novo red patch was 45 years, with an increasing trend in the proportion of malignant red patches with age, especially in patients over 60 years.

There is a paucity of evidence regarding whether more experienced and senior clinicians have better discrimination of malignancy in red patches or better diagnostic outcomes with cystoscopy. One small study on 50 patients in a nurse‐led cystoscopy clinic showed good agreement between a specialist urology nurse and urology registrar/trainee in diagnosis and management, suggesting clinical role is not as important as experience.^9^ Another study assessing competency in flexible cystoscopy by a single urology trainee with no previous experience showed an acceptable performance by the 122nd procedure, but complete competence was achieved following 289 procedures.^10^ Whilst the lack of statistically significant difference in outcomes between experience levels in this study is consistent with previous work, this may be due to a lack of power as the confidence intervals were wide.

The strengths of this study are in its large sample size of biopsied isolated de novo red patches, and sub‐analyses to give better granularity and understanding of malignancy in these red patches. Additionally, its diverse population increases the generalisability of the findings. To our knowledge, we are the first study to consider the difference between clinician‐visually reported suspicious and non‐suspicious red patches and explore the association of well‐known patient risk factors with malignancy in red patches.

The main limitation of this analysis is that as an observational study, not all patients underwent a biopsy thereby introducing selection bias and possible underpowering in the analysis and multivariable model, though the results are biologically plausible, and it would not have been clinically justified to perform biopsy in all patients with a red patch if the index of suspicion was low. Furthermore, the full scope of outcomes might not be adequately represented due to selection bias. Data was not collected on reasons not to biopsy, but this could have been due to patient consent, co‐morbidity or frailty or conservative management with follow‐up to assess if the red patch resolves. Additionally, we did not measure or adjust for inter‐operator variability in suspicious and non‐suspicious reported red patches, which may influence the accuracy and consistency of results. The subjectivity in the red patch classification of suspicious and non‐suspicious may lead to further inconsistent results as it is not an objective standardised classification. Finally, not all patients had cytology, reducing its ability to assess the full diagnostic value of cytology in identifying malignancy.

As the analysis excludes patients with previous bladder cancer or concomitant red patches alongside a suspected bladder cancer, the results should not be extrapolated to all red patches, only isolated de novo red patches in patients with suspected urinary tract cancer and no prior history of bladder cancer.

## 5 CONCLUSIONS

Our results suggest that the clinician's suspicion of a de novo red patch being malignant is important. Red patches deemed non‐suspicious have a low malignancy rate and may avoid biopsy in select cases in favour of surveillance. The clinician may consider the patient's risk factors of age and smoking, and non‐invasive tests such as cytology before deciding on a biopsy, which carries risks of bleeding, infection and perforation.

### 5.1 Declaration of Generative AI and AI‐assisted technologies in the writing process

During the preparation and writing of the work, the authors did not use any generative AI or AI‐assisted technologies.

## AUTHOR CONTRIBUTIONS

Sinan Khadhouri and John S. McGrath were responsible for the study idea. Sinan Khadhouri, Veeru Kasivisvanathan and Taimur T. Shah developed the concept. Sinan Khadhouri, Kevin, Gallagher, Taimur T. Shah and Veeru Kasivisvanathan were responsible for the study design. Sinan Khadhouri, Kevin Gallagher and Kenneth R. MacKenzie were responsible for coordinating the study. Sinan Khadhouri, Kenneth R. MacKenzie, Taimur T. Shah, Chuanyu Gao and Eleanor Zimmermann were responsible for data quality assurance. Sinan Khadhouri and Kevin Gallagher were involved in data cleaning and statistical analysis. Sinan Khadhouri wrote the first draft of the manuscript. All mainline authors were involved in the interpretation, editing, critical review and final approval of the manuscript. All collaborators were involved in data collection.

## CONFLICT OF INTEREST STATEMENT

None to declare.
